# Association of Socioeconomic Position and Demographic Characteristics with Cardiovascular Disease Risk Factors and Healthcare Access among Adults Living in Pohnpei, Federated States of Micronesia

**DOI:** 10.1155/2014/595678

**Published:** 2014-12-22

**Authors:** G. M. Hosey, M. Samo, E. W. Gregg, L. Barker, D. Padden, S. G. Bibb

**Affiliations:** ^1^Division of Diabetes Translation, Centers for Disease Control and Prevention, National Center for Chronic Disease and Health Promotion, 4770 Buford Highway, Mailstop K10, Atlanta, GA 30341, USA; ^2^Department of Health and Social Affairs, Federated States of Micronesia National Government, P.O. Box PS70, Palikir, FM 96941, USA; ^3^Daniel K. Inouye Graduate School of Nursing, Uniformed Services University of the Health Sciences, 4301 Jones Bridge Road, Bethesda, MD 20814, USA

## Abstract

*Background*. The burden of cardiovascular disease (CVD) is increasing in low-to-middle income countries. We examined how socioeconomic and demographic characteristics may be associated with CVD risk factors and healthcare access in such countries. *Methods*. We extracted data from the World Health Organization's STEPwise approach to surveillance 2002 cross-sectional dataset from Pohnpei, Federated States of Micronesia (FSM). We used these data to estimate associations for socioeconomic position (education, income, and employment) and demographics (age, sex, and urban/rural) with CVD risk factors and with healthcare access, among a sample of 1638 adults (25–64 years). *Results*. In general, we found significantly higher proportions of daily tobacco use among men than women and respondents reporting primary-level education (<9 years) than among those with postsecondary education (>12 years). Results also revealed significant positive associations between paid employment and waist circumference and systolic blood pressure. Healthcare access did not differ significantly by socioeconomic position. Women reported significantly higher mean waist circumference than men. *Conclusion*. Our results suggest that socioeconomic position and demographic characteristics impact CVD risk factors and healthcare access in FSM. This understanding may help decision-makers tailor population-level policies and programs. The 2002 Pohnpei data provides a baseline; subsequent population health surveillance data might define trends.

## 1. Introduction

Population-based surveillance—the ongoing systematic collection, analysis, and interpretation of health data—is critical for providing information on which to base policy; prioritize resources; guide program planning, evaluation, and research; and protect and promote population health [1]. The World Bank defines lower to middle income countries (LMICs) as countries where residents have mean income less than $12,615 (2012, http://data.worldbank.org/about/country-classifications). In LMIC, the burden of cardiovascular disease (CVD) is increasing at faster rates than those experienced by high-income countries in previous decades, elevating the need to strengthen country-level surveillance [2, 3].

Worldwide, over 80% of CVD deaths occur in LMICs [4]. Additionally, in LMICs, 29% of deaths from chronic noncommunicable diseases occur before the age of 60 years, compared to 13% in high-income countries [4]. Research evidence, primarily from high-income countries, shows an inverse association for indicators of socioeconomic position, measured by education, income, or employment status, with CVD risk factors [5, 6]. Although evidence is limited, most LMIC might follow a similar pattern [7, 8]. Providing better evidence on the impact of socioeconomic position on CVD risk factors in LMICs may help decision-makers tailor policy and programmatic interventions to fit conditions in these countries.

**Figure 1:**
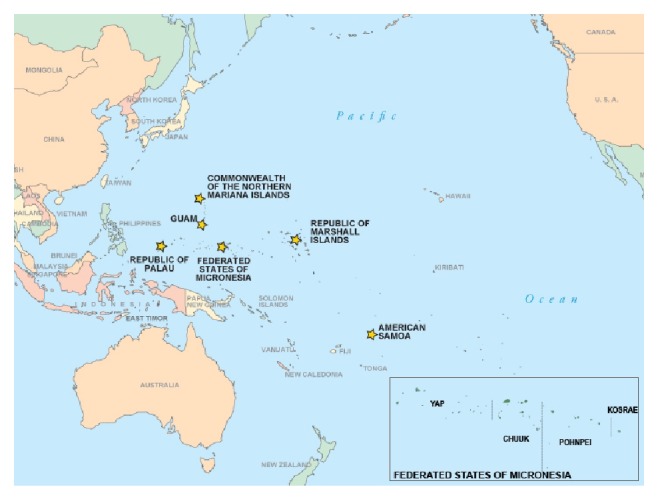
Map of the US Associated Pacific Island Jurisdictions, Federated States of Micronesia.

In 2002, Pohnpei State, Federated States of Micronesia (FSM) (Figure 1), implemented a population-based surveillance survey to measure chronic disease risk factors among adults. While survey reports provide weighted analysis as a whole and by subgroup (i.e., age and sex), disaggregated analysis by socioeconomic position is unavailable [9]. In an effort to increase understanding of CVD risk factors within the population, the FSM Department of Health and Social Affairs requested assistance in broadening the analysis of the available population-based dataset.

This study describes the exploratory analysis of the association between socioeconomic position, measured by education, income, and employment status with CVD risk factors (i.e., behavioral and anthropometric/biochemical measures) and healthcare access among adults living in Pohnpei State, FSM.

## 2. Methods

### 2.1. Identifying the Study Sample

The STEPwise approach to surveillance (STEPS) is a population-based, cross-sectional survey of adults. Detailed STEPS design and methodology are available at http://www.who.int/chp/steps/en/. The STEPS data collection process, developed and administered by the World Health Organization, includes three sequential steps: (1) personal interview, (2) anthropometric measurements, and (3) biochemical assessment. After training, field staff collected data using standardized procedures and protocols.

We conducted a descriptive, cross-sectional secondary analysis of the 2002 dataset from the FSM (Pohnpei) STEPS [9]. The 2002 STEPS used a multistage, probabilistic, and cluster design (based on 2000 FSM Pohnpei census enumeration areas) to randomly select households for participation. Data were obtained from 1638 adults aged 25–64 years, with a 78% response rate. To ensure population representativeness, the created sample for this study included the entire 2002 STEPS Pohnpei dataset [9].

### 2.2. Ethical Considerations

Prior to our downloading STEPS data, the FSM Department of Health and Social Affairs and the Uniformed Services University Institutional Review Board approved this study.

### 2.3. Variables Used for Analyses

#### 2.3.1. Socioeconomic Position

We used self-reported educational attainment, estimated annual household income, and employment status as indicators of socioeconomic position.

*Education*. Education is a widely used indicator of socioeconomic position, as formal education is usually completed by early adulthood and therefore remains stable across the adult lifespan. Additionally, education is a key determinant of a person's employment and income potential and for developing life-skills making him or her more likely to adopt health-promoting behaviors [6]. Using years of schooling reported and FSM education system levels, we categorized study participants into one of three groups based on educational attainment: <9 years (primary), 9–12 years (secondary), or >12 years (postsecondary).

Income and employment status are also widely used as indicators of socioeconomic position as each can provide access to health-promotion resources (through ability to pay or employer-provided insurance) that can contribute toward better health outcomes [6]. 

*Income*. We categorized study participants into one of three groups by estimated annual household income: <$5,000, $5,000–$10,000, or >$10,000. We added a category, “unknown income,” to account for missing values for some participants. 

*Employment Status*. We categorized study participants into one of three groups by employment status: paid (government job, nongovernment job, or self-employed); unpaid (retiree, volunteer, student, homemaker, or subsistence); or unemployed.

#### 2.3.2. Covariates

We included sex, age, and place of residence as covariates. Sex and age may impact the association between socioeconomic position and CVD risk factors, mirroring variations in both biological and social influences across societies [6, 10]. We categorized study participants into one of four age groups: 25–34 years, 35–44 years, 45–54 years, or 55–64 years. Some evidence suggests that urban populations in LMICs have higher CVD risk than rural populations in those countries. This greater risk may be attributable to lifestyle changes in urban populations [4]. Using census enumeration codes, we categorized study participants by place of residence as either urban or rural, defining* urban* as residing in one of two developed municipalities within Pohnpei.

#### 2.3.3. Behavioral Risk Factors

Modifiable behavioral risk factors known to increase CVD risk include tobacco use, inadequate fruit and vegetable consumption, and physical inactivity [4]. Using information on these factors available in the STEPS dataset, we created three dichotomized (yes/no) behavioral variables: (1) “daily tobacco,” defined as using at least one smoke or smokeless tobacco product per day; (2) “five servings/day,” defined as mean total fruit and vegetable intake of at least five servings per day (typical week); and (3) “active,” which we determined using a computed score that accounted for intensity (moderate or vigorous), duration, or metabolic rate equal to at least thirty minutes of moderate physical activity five days a week.

#### 2.3.4. Healthcare Access

Socioeconomic position is also linked to healthcare access, which can be measured by potential (i.e., availability of healthcare services) or realized measures (i.e., use of services) [11]. Because specific measures of healthcare access were not available in the dataset, we created a proxy yes/no measure of “access,” based on participant self-report of having been screened for elevated blood pressure (BP) or blood glucose or having been told by a doctor or other healthcare professionals that he or she had elevated BP within the past year.

#### 2.3.5. Anthropometric/Biochemical Risk Factors

We used direct physical and biochemical measures that are strongly associated with CVD risk and were available in the dataset: BP, height, weight, waist circumference, fasting blood glucose, and fasting blood lipids [4]. Detailed methodology is available at http://www.who.int/chp/steps/en/.

*Blood Pressure*. We used the mean of the two most recent BP measurements to create systolic and diastolic BP variables. We defined high BP as ≥140 mmHg for systolic and ≥90 mmHg for diastolic or hypertension medication taken within last two weeks or self-report of hypertension diagnosis within the last 12 months [12]. 

*BMI and Waist Circumference*. We included body mass index (BMI) and waist circumference as obesity variables, categorizing obesity as BMI ≥ 30 kg/m^2^ and central (abdominal) obesity as a waist circumference of >94 cm for men and >88 cm for women [13]. 

*Diabetes and Blood Lipids*. We defined diabetes as fasting blood glucose ≥126 mg/dL [14] or current diabetes treatment (i.e., insulin or hypoglycemic agent taken within last two weeks) or self-report of diabetes diagnosis; high cholesterol as a fasting cholesterol ≥200 mg/dL; high triglyceride as a fasting triglyceride ≥150 mg/dL; and low HDL (high-density lipoprotein) as a fasting HDL <40 mg/dL for men and <50 mg/dL for women [15].

Variables for obesity and diabetes were not included for pregnant women.

### 2.4. Statistical Methods

We analyzed the data using SPSS version 20.0 complex samples module that accounts for the complex sampling design used in the STEPS survey, correctly calculating standard errors with weighted data. We applied sex-age structure survey weights (standardized to the FSM 2000 census for Pohnpei) to provide results representative of the adult Pohnpeian population aged 25–64 years. After data cleaning and recoding, we completed descriptive analysis for all variables. Our analysis included chi-square with Rao-Scott adjustment and one-way analysis of variance with post hoc pairwise comparisons, using Bonferroni adjustment criterion, to determine the associations between socioeconomic position and demographic characteristics with selected CVD risk factors and healthcare access. Mean fruit and vegetable consumption and fasting blood glucose were excluded from the analysis of variance. This was because examination of normal Q-Q plots showed that residuals for these variables were not normally distributed, thereby violating the assumptions required for analysis of variance. We used an alpha level of 0.05 to represent significance for all statistical tests.

## 3. Results

Here we present some key findings from the study.  Table 1 illustrates selected characteristics from the sample dataset, collectively and stratified by sex, for the overall population. Mean age for all respondents was 39.7 years. Most respondents reported a primary-level education (<9 years) and incomes <$5,000. More than one-quarter reported daily tobacco use, while less than one-fifth consumed five or more servings of fruit and vegetables per day or engaged in the recommended physical activity. Compared to males, female respondents had a higher percentage of central obesity (39% versus 85%, resp.) and raised fasting blood glucose (26.8% versus, 37.7%, resp.).

Table 2 shows significant associations between socioeconomic position and demographic characteristics with selected CVD risk factors and healthcare access based on chi-square analysis. For example, analysis indicated the following.

- Persons with a primary-level education had significantly higher rates of daily tobacco use and physical activity than those with higher educational attainment. Persons with annual income <$5,000 had significantly higher rates of daily tobacco use than other income groups. A significantly higher proportion of persons with paid employment reported daily tobacco use than did unpaid and unemployed respondents.
- Men reported significantly higher rates of daily tobacco use and physical activity than women.
- A significantly higher proportion of young-to-middle-aged (25–44 years) respondents reported daily tobacco use than older age groups.

Table 3 provides estimated marginal means and standard errors from the analysis of variance for socioeconomic position, sex, age, and place of residence and continuous variables (age-sex standardized). Using one-way analysis of variance with post hoc comparisons, we found the following.

- Persons with incomes greater than $10,000 had significantly higher systolic BP than those with incomes <$5,000 (129.9 mmHg, CI = 127.0–132.8 versus 122.2 mmHg, CI = 120.2–124.1, *P* < 0.001).
- Unemployed persons had a smaller mean waist circumference than those with paid employment (91.8 cm, CI = 89.7–93.8 versus 94.6 cm, CI = 93.2–96.0, *P* = 0.011).
- Women had significantly higher mean BMI (31.3 kg/m^2^, CI = 30.7–32.0 versus 27.8 kg/m^2^, CI = 27.1–28.5 in men, *P* < 0.001) and greater mean waist circumference (95.2 cm, CI = 93.5–96.9 versus 91.5 cm, CI = 89.9–93.1, *P* < 0.001) than men.

## 4. Discussion

Although CVD rates are declining in high-income countries, LMICs are experiencing an increasing burden, particularly among those aged <60 years [4]. Evidence from our study supports the use of quality population-based secondary data to better understand and provide useful information for developing CVD prevention efforts in LMICs.

### 4.1. Comparisons with Findings from Other Studies

Similar to other studies, our analysis showed higher rates of daily tobacco use in men compared to women. For example, in 2009, an estimated 36% of men worldwide (aged >15 years) smoked, compared to fewer than 8% of women [16]. Recent studies analyzing LMIC data from the World Health Survey [17] and the Global Adult Tobacco Survey [18] also have found that tobacco use disproportionately affects men. We also found that low educational attainment was significantly associated with higher rates of daily tobacco use, while annual household income was inversely associated. An analysis of World Health Survey data (representing 48 LMICs) also found that, among all adults, low education was strongly associated with higher rates of smoking and, for low-income countries, wealth was inversely associated with smoking [17].

While our analysis showed no evidence that BMI was significantly associated with socioeconomic position, we found several positive associations for waist circumference with socioeconomic position and selected demographic characteristics. Our results support those from other studies suggesting that measures of central obesity may be an equally or more relevant measure for predicting obesity-related health risks than BMI [19, 20].

We found that education was inversely associated with HDL, income was positively associated with higher BP, and increased age was positively associated with total cholesterol; some other studies in LMICs have reported different results for these associations. For example, in rural Vietnamese adults, aged 25–64 years, hypertension was inversely associated with education level [21]. Another study among urban Latin American adults aged ≥18 years found inverse associations for income and education with hypertension and for education with diabetes [22]. Additionally a study, among adults in Brazil aged ≥20 years, examined cluster measures of CVD risk factors and found inverse associations for those risk factors with education in both sexes and with income in men [23].

For high-income countries, studies have documented a transition, through the progression of socioeconomic development, from a direct to an inverse association between socioeconomic position and CVD risk factors [5, 6]. Researchers have suggested that this transition occurs because the wealthier and more educated persons tend to be early adopters of high-risk behaviors that contribute to CVD. As the wealthy realize the health-related consequences, they become early adopters of lifestyle change and disease prevention, subsequently lowering their CVD risk [24, 25]. In comparison, the uneducated poor may experience later peaks in the CVD risk factors and carry a higher burden of CVD after rates decline among the wealthy [24]. It is expected that LMIC will follow a similar social pattern, as economies develop [5, 6].

While evidence is limited, the varied patterning among socioeconomic position and CVD risk factors in our study might suggest a gradual shift in the epidemiologic transition within Pohnpei. For example, seminal cross-sectional studies conducted in Pohnpei (1947 and 1953) found low BP among adults, aged 20–60 years, while a 25-year follow-up study reported significantly increased diastolic BP among urban males (20–60 years) living in Kolonia, Pohnpei, attributed to increased urbanization [26]. Another review of research studies completed in Western and American Samoa, between 1982 and 2003, observed that, as country-level development improved, the burden of obesity shifts to lower socioeconomic groups [27]. Recognizing the complex dynamics involved in the epidemiologic transition within LMIC, further research is needed to examine the trends in the socioeconomic patterning of CVD risk over time.

While about one-half (48%) of respondents in our study reported annual household income <$5,000, we found no associations between income and healthcare access. Other studies have reported that low-income groups are more likely to be uninsured and less likely to seek healthcare, including screening and treatment [28–30]. A possible explanation for the positive association between rural residence and healthcare access found by our study may involve targeted rural outreach and health screenings offered at no cost through the FSM Department of Health and Social Affairs and other community agencies.

### 4.2. Strengths and Limitations

We recognize that the analysis of healthcare access in our study addressed only proxy measures of realized healthcare services [11]. Further research would be required if we are to better understand the more complex issues related to healthcare access in this population (i.e., cultural norms, usual providers, insurance coverage, and unmet medical needs). A limitation of our study was use of self-reported responses from the 2002 Pohnpei STEPS to assess behavioral risk measures, which are subject to participant recall, social desirability, and response bias. The limited data collection period, within the primary study, may have also introduced seasonal variation in responses. These potential biases could contribute to under- or overreporting of risk factors [31].

While the 2002 Pohnpei STEPS data provides a baseline for the association between socioeconomic position and CVD risk factors, data from subsequent STEPS surveys are needed to provide reliable information on trends in these associations over time. For example, since 2004, FSM Department of Health and Social Affairs, in partnership with community networks, has promoted physical activity, consumption of local fruit and vegetables, and tobacco-free living, through awareness campaigns and policy development [32, 33]. Data from subsequent STEPS surveys and analysis might reveal distinct reversals of CVD risk factor patterns found in this study.

Inherent methodological biases in our study model limit the generalizability of findings beyond Pohnpei and other subpopulations in FSM. For example, because the STEPS dataset did not provide probability variables for sample selection, we assigned standardized age-sex rates, which may not have been representative of the 2002 adult Pohnpeian population. Additionally, the cross-sectional data we used did not allow interpretation of causal relationships. Finally, CVD risk factors were analyzed individually. However, a majority of respondents (52.6%) reported 3–5 risk factors [9]. This clustering of cardiovascular disease risk factors increases the risk of CVD morbidity/mortality [34, 35]. Using risk factor cluster analysis might help further identify population segments that would benefit from policy and programmatic interventions to reduce CVD morbidity and mortality.

The strengths of our study include using a large population-based dataset and objective anthropometric and biochemical measures and the collaboration with the FSM Department of Health and Social Affairs leadership throughout the study. The intent of the collaborations with FSM leadership was to integrate the knowledge and insight obtained from this study in supporting population-level policies and programs targeting the reduction of CVD risk factors in country. Our study also supports secondary analysis as an efficient methodology for building the basis for population health research within FSM.

## 5. Conclusion

Overall, our results suggest that socioeconomic position has an impact on CVD risk factors among adults living in Pohnpei. This understanding may help decision-makers tailor population-level policies and programs for residents of FSM. While the 2002 Pohnpei STEPS data provides a baseline for the association between socioeconomic position and CVD risk factors, further research is needed to expand the scientific evidence between socioeconomic position and demographic characteristics with CVD risk factors and healthcare access operating within LMIC.

## Figures and Tables

**Table 1:** Selected characteristics of study sample using the 2002 STEPS FSM (Pohnpei) dataset^a^.

Characteristic	Male	Female	Total sample

*N*	642	996	1638
Age mean (95% CI)	39.8 (38.9–40.7)	39.5 (38.7–40.3)	39.7 (39.0–40.3)

Characteristic (measure)	*n* (% of males)	*n* (% of females)	*n* (% of total)

Education (highest level completed)			
Primary (<9 y)	315 (52.2)	568 (59.1)	883 (55.6)
Secondary (9–12 y)	183 (31.4)	302 (34.1)	485 (32.7)
Postsecondary (≥13 y)	99 (16.4)	57 (06.8)	156 (11.6)
Estimated annual household income			
Low (<$5,000)	298 (47.5)	486 (49.3)	784 (48.4)
Middle ($5,000–$10,000)	121 (18.4)	170 (16.8)	291 (17.6)
High (>$10,000)	65 (09.1)	93 (09.1)	158 (09.1)
Unknown	158 (25.0)	247 (24.7)	405 (24.9)
Employment status^b^			
Paid (NA)	409 (67.7)	324 (36.1)	733 (52.3)
Unpaid (NA)	66 (09.8)	267 (29.2)	333 (19.3)
Unemployed (NA)	125 (22.5)	313 (34.6)	438 (28.4)
Behavioral^c^			
Daily tobacco use (NA)	235 (37.7)	169 (16.7)	404 (27.3)
Fruit/vegetables (≥5 servings/day)	119 (18.7)	179 (17.6)	298 (18.2)
Physically active (≥30 min/d, 5 d/wk)	140 (27.4)	94 (11.4)	234 (19.6)
Healthcare access (NA)^d^	67 (09.5)	141 (13.9)	208 (11.7)
Anthropometric & biochemical			
Overweight (BMI ≥ 25 kg/m^2^ <30kg/m^2^)	201 (33.8)	237 (26.6)	438 (30.3)
Obesity (BMI ≥ 30 kg/m^2^)	187 (30.1)	503 (55.9)	690 (42.7)
Central obesity (men > 94 cm; women > 80 cm)	265 (39.0)	794 (85.0)	1059 (61.3)
High BP (≥140/90 mmHg, use of BP medication in last two wk,	209 (29.3)	209 (19.2)	418 (24.3)
or self-report of HTN diagnosis in last y)			
Diabetes (≥126 mg/dL, use of insulin or hypoglycemic agent in last	46 (26.8)	111 (37.7)	157 (32.6)
two wk, or self-report of diabetes diagnosis in last y)			
High total cholesterol (≥200 mg/dL)	81 (50.4)	117 (44.5)	198 (47.4)
High triglyceride (≥150 mg/dL)	30 (22.5)	36 (15.3)	66 (18.8)
Low HDL (<40 mg/dL [men]; <50 mg/dL [women])	78 (53.5)	199 (86.4)	277 (70.0)

CI: confidence interval; y: year(s); d: day(s); wk: week(s); min: minute(s); BP: blood pressure; HTN: hypertension; FBG: fasting blood glucose; cm: centimeter(s); mg: milligram(s); dL: deciliter(s); mmHg: millimeters of mercury; HDL: high-density lipoprotein cholesterol; *N*: size of population; *n*: size of sample; NA: not applicable.

^a^All estimates are age/sex standardized to the FSM 2000 Pohnpei census. Behavioral variables are self-report; anthropometric and biochemical variables are direct measures; biochemical variables exclude nonfasting values; obesity and FBG exclude pregnant women.

^b^“Paid” category includes government, nongovernment, or self-employed; “unpaid” category includes retiree, volunteer, student, homemaker, or subsistence.

^c^Daily tobacco use includes daily use of cigarettes, cigars, pipes, or smokeless tobacco; fruit/vegetable consumption includes at least five servings fruit or vegetables/day; physically active includes ≥30 min/day moderate activity, ≥5 days/wk or ≥3 days vigorous activity (>20 min/day, or ≥600 metabolic equivalent of task-min/wk).

^d^Healthcare access defined as a blood glucose or BP screening or HTN diagnosis in last year.

**Table 2:** Prevalence of selected behavioral and healthcare access measures by socioeconomic and other characteristics using the 2002 STEPS FSM (Pohnpei) dataset^a^.

Characteristic	Daily tobacco use^b^				Physically active^c^				Healthcare access^d^			
	*n*	% (95% CI)	*F* (df1, df2)	*P*	*n*	% (95% CI)	*F* (df1, df2)	*P*	*n*	% (95% CI)	*F* (df1, df2)	*P*
Education												
Primary (<9 y)	243	62.1 (54.5–69.1)	5.01 (1.7, 49.2)	0.012	119	50.0 (41.1–58.9)	5.87 (1.7, 49.6)	0.007	99	48.0 (43.5–52.6)	2.56 (1.6, 45.5)	0.101
Secondary (9–12 y)	108	29.3 (23.7–35.7)			71	32.2 (26.4–38.5)			71	38.9 (32.5–45.8)		
Postsecondary (≥13 y)	30	08.6 (05.8–12.5)			34	17.8 (12.1–25.4)			24	13.1 (08.1–20.3)		
Income												
<$5,000	210	53.0 (44.0–61.9)	3.70 (2.6, 75.9)	0.020	127	54.9 (46.0–63.6)	2.14 (2.8, 80.8)	0.107	85	41.8 (33.7–50.3)	2.28 (2.6, 76.8)	0.093
$5,000–$10,000	61	14.7 (10.2–20.7)			45	18.9 (13.5–25.9)			44	20.3 (15.0–26.9)		
>$10,000	24	05.3 (03.3–08.5)			19	06.7 (03.6–12.2)			30	13.3 (08.9–19.3)		
Employment status^e^												
Paid	215	59.6 (52.6–66.2)	6.33 (1.9, 55.4)	0.004	121	56.7 (48.5–64.5)	3.30 (2, 57)	0.045	109	59.5 (52.1–66.4)	3.25 (1.9, 56.5)	0.047
Unpaid	69	15.3 (12.0–19.5)			34	12.8 (08.7–18.4)			40	19.5 (15.3–24.4)		
Unemployed	96	25.0 (20.2–30.6)			67	30.6 (23.0–39.4)			45	21.1 (15.1–28.6)		
Sex												
Male	235	69.6 (64.5–74.2)	64.97 (1, 29)	0.000	140	71.8 (66.0–76.9)	59.91 (1, 29)	0.000	67	41.0 (34.0–48.4)	7.35 (1, 29)	0.011
Female	169	30.4 (25.8–35.5)			94	28.2 (23.1–34.0)			141	59.0 (51.6–66.0)		
Place of residence												
Urban	82	19.2 (08.8–36.9)	3.12 (1, 29)	0.088	50	20.5 (09.0–40.1)	0.01 (1, 29)	0.921	71	34.4 (17.3–56.7)	10.45 (1, 29)	0.003
Rural	322	80.8 (63.1–91.2)			184	79.5 (59.9–91.0)			137	65.6 (43.3–82.7)		
Age												
25–34 y	105	33.1 (28.6–37.9)	9.45 (2.8, 81.7)	0.000	83	44.4 (37.9–51.2)	2.89 (2.5, 71.3)	0.051	54	33.8 (26.8–41.7)	3.46 (2.7, 78.4)	0.024
35–44 y	146	39.0 (33.7–44.5)			75	33.4 (26.5–41.2)			54	26.5 (20.3–33.9)		
45–54 y	118	22.0 (18.1–26.3)			50	14.7 (11.3–18.8)			66	25.8 (20.8–31.4)		
55–64 y	35	06.0 (04.1–08.7)			26	07.5 (05.2–10.7)			34	13.8 (09.8–19.2)		

y: years; *n*: sample size; *F*: Rao-Scott adjustment to the *χ* ^2^ for complex samples (e.g., adjusted *F*); df: degrees of freedom.

^a^All estimates are age/sex standardized to the FSM 2000 Pohnpei census; *P* values are based on the adjusted *F* and its degrees of freedom.

^b^Daily tobacco use defined as self-report of daily use of cigarettes, cigars, pipes, or smokeless tobacco product.

^c^Physically active includes ≥30 min/d of moderate activity, ≥5 d/wk or ≥3 d of vigorous activity (>20 min/d), or ≥600 metabolic equivalent of task-min/wk.

^d^Healthcare access defined as self-reported screening for blood pressure or blood glucose of hypertension diagnosis in the last year.

^e^“Paid” category defined as government, nongovernment, or self-employed; “unpaid” category defined as retiree, volunteer, student, homemaker, or subsistence.

**Table 3:** One-way analysis of variance estimated marginal means and standard errors for socioeconomic and demographic characteristics and selected CVD risk factors using the 2002 STEPS FSM (Pohnpei) dataset^a^.

Characteristic	BMI (kg/m^2^)	Waist (cm)	SBP (mmHg)	DBP (mmHg)	Total chol (mg/dL)	Triglyc (mg/dL)	HDL (mg/dL)
Education (highest level completed)							
Primary (<9 y)	29.3 (0.3)	92.8 (0.8)	125.0 (1.0)	75.6 (0.6)	201.4 (4.1)	113.1 (8.8)	41.6 (1.4)
Secondary (9–12 y)	30.0 (0.4)	94.0 (0.9)	122.4 (0.9)	74.4 (0.5)	194.5 (6.0)	106.0 (7.2)	40.3 (1.6)
Postsecondary (≥13 y)	29.1 (0.8)	92.4 (1.1)	126.7 (1.3)	76.8 (0.8)	202.7 (5.8)	106.0 (9.4)	38.7 (1.2)
Estimated annual household income							
<$5,000	29.1 (0.4)	92.0 (0.9)	122.2 (1.0)	74.6 (0.5)	197.2 (6.2)	103.6 (9.0)	41.6 (2.1)
$5,000–$10,000	30.0 (0.4)	94.5 (1.2)	124.5 (1.0)	74.5 (0.6)	202.4 (5.0)	125.2 (9.2)	40.2 (1.4)
>$10,000	30.2 (0.6)	96.6 (1.3)	129.9 (1.4)	78.3 (1.1)	199.4 (5.2)	89.5 (6.3)	39.1 (1.5)
Employment status^b^							
Paid	29.7 (0.3)	94.6 (0.7)	126.2 (0.7)	76.5 (0.5)	203.7 (4.6)	113.6 (7.4)	41.3 (2.0)
Unpaid	29.4 (0.6)	93.2 (1.3)	122.0 (1.4)	73.6 (0.7)	190.8 (5.5)	91.1 (7.7)	40.0 (1.2)
Unemployed	29.5 (0.5)	91.8 (1.0)	122.0 (1.3)	74.4 (0.7)	193.6 (4.0)	107.7 (7.7)	40.3 (1.5)
Sex							
Male	27.8 (0.3)	91.5 (0.8)	129.6 (0.9)	77.6 (0.6)	202.3 (4.6)	119.5 (6.0)	40.8 (1.9)
Female	31.3 (0.3)	95.2 (0.8)	118.7 (1.0)	73.1 (0.5)	196.5 (3.9)	99.4 (7.2)	40.6 (0.9)
Age group							
25–34 y	29.2 (0.4)	90.2 (0.9)	119.5 (0.9)	72.2 (0.6)	184.5 (6.2)	95.2 (7.2)	40.4 (1.4)
35–44 y	29.5 (0.5)	93.2 (1.0)	122.7 (1.1)	75.7 (0.8)	196.4 (5.1)	107.7 (9.4)	41.0 (2.2)
45–54 y	30.0 (0.3)	97.4 (0.9)	130.2 (1.2)	79.1 (0.7)	209.1 (3.3)	125.0 (14.6)	40.2 (0.9)
55–64 y	29.9 (0.7)	97.1 (1.3)	135.2 (2.3)	78.2 (1.0)	229.9 (4.0)	117.5 (7.6)	42.3 (1.3)
Place of residence							
Urban	30.2 (0.5)	96.4 (1.3)	124.7 (1.4)	75.3 (0.7)	198.4 (5.0)	103.2 (2.4)	37.9 (1.9)
Rural	29.3 (0.3)	92.4 (0.8)	124.0 (0.9)	75.3 (0.5)	199.7 (4.1)	111.1 (6.3)	41.8 (1.5)

CVD: cardiovascular disease; BMI: body mass index; kg/m^2^: kilograms per meter squared; cm: centimeters; SBP: systolic blood pressure; mmHg: millimeters of mercury; DBP: diastolic blood pressure; Chol: cholesterol; Triglyc: triglycerides; HDL: high-density lipoprotein; y: year(s).

^a^All estimates are age/sex standardized to the FSM 2000 Pohnpei census. Anthropometric and biochemical direct measures; biochemical and obesity measures exclude nonfasting.

^b^Paid category includes government, nongovernment, or self-employed; unpaid category includes retiree, volunteer, student, homemaker, or subsistence.
